# Psychosocial screening and assessment in oncology and palliative care settings

**DOI:** 10.3389/fpsyg.2014.01485

**Published:** 2015-01-07

**Authors:** Luigi Grassi, Rosangela Caruso, Silvana Sabato, Sara Massarenti, Maria G. Nanni, the UniFe Psychiatry Working Group Coauthors

**Affiliations:** ^1^Clinical Psychiatry, Department of Biomedical and Specialty Surgical Sciences, University of FerraraFerrara, Italy; ^2^University Hospital Psychiatric Unit, Program of Psycho-Oncology and Psychiatry in Palliative Care Integrated Department of Mental Health and Drugs Abuse, S. Anna University Hospital and Health Authority FerraraItaly

**Keywords:** psychosocial dimensions, DSM5, DCPR, psycho-oncology, cancer

## Abstract

Psychiatric and psychosocial disorders among cancer patients have been reported as a major consequence of the disease and treatment. The problems in applying a pure psychiatric approach have determined the need for structuring more defined methods, including screening for distress and emotional symptoms and a more specific psychosocial assessment, to warrant proper care to cancer patients with psychosocial problems. This review examines some of the most significant issues related to these two steps, screening and assessment of psychosocial morbidity in cancer and palliative care. With regard to this, the many different variables, such as the factors affecting individual vulnerability (e.g., life events, chronic stress and allostatic load, well-being, and health attitudes) and the psychosocial correlates of medical disease (e.g., psychiatric disturbances, psychological symptoms, illness behavior, and quality of life) which are possibly implicated not only in “classical” psychiatric disorders but more broadly in psychosocial suffering. Multidimensional tools [e.g., and specific psychosocially oriented interview (e.g., the Diagnostic Criteria for Psychosomatic Research)] represent a way to screen for and assess emotional distress, anxiety and depression, maladaptive coping, dysfunctional attachment, as well as other significant psychosocial dimensions secondary to cancer, such as demoralization and health anxiety. Cross-cultural issues, such as language, ethnicity, race, and religion, are also discussed as possible factors influencing the patients and families perception of illness, coping mechanisms, psychological response to a cancer diagnosis.

## INTRODUCTION

Psychiatric and psychosocial disorders among cancer patients have been reported as a major consequence of the disease and treatment. Sutherland (1956) among the first indicated that the psychology of cancer patient is that of a person under a special and severe form of stress, during which many fundamental underlying convictions, based on the life-history of the person and his experiences (e.g., pattern of relationship with attachment figures) are brought to the surface. He also described six clinical types of psychological reactions commonly seen after cancer diagnosis and treatment, namely dependency, anxiety, postoperative depression, hypochondriac response, obsessive-compulsive reactions, and paranoid reactions.

Subsequent research, by using more specific tools and interviews (e.g., the Diagnostic Statistical Manual for Mental Disorders DSM-III, III-R, and IV; the International Classification of Disorders), has confirmed the importance of assessing psychosocial responses, indicating that adjustment disorders, anxiety, and depression may be diagnosed in between 40–50% of cancer patients (Derogatis et al., 1983; Hardman et al., 1989; Grassi et al., 2000; Kissane et al., 2004; Singer et al., 2013). The implications and the impact of these disorders for the patients and the families are of paramount importance in oncology, with studies demonstrating the association of psychosocial morbidity with maladaptive coping, reduction of quality of life (QoL), impairment in social relationships, risk of suicide, longer rehabilitation time, poor adherence to treatment, and abnormal illness behavior, family dysfunction, and possibly, shorter survival (Mitchell et al., 2011).

The problems in applying a “pure” psychiatric approach, in conducing structured psychiatric interviews in cancer settings (Grassi and Nanni, 2013) and in having the largest possible number of cancer patients evaluated in their psychosocial dimension, according to the new standard of treatment (Holland et al., 2011) have determined the need for structuring more defined methods. There is today a general agreement that screening for distress and emotional symptoms is the first important procedure to be implemented in clinical settings, while a more specific psychosocial assessment should follow, to warrant proper care to cancer patients with psychosocial problems (Grassi et al., 2013a). This review examines some of the most significant issues related to these two steps, screening and assessment of psychosocial morbidity in cancer and palliative care.

## SCREENING FOR EMOTIONAL DISORDERS

In oncology and palliative care settings there are several dimensions including emotional distress, anxiety and depression, maladaptive coping, and dysfunctional attachment that, a broad psychosomatic approach can elicit (Grassi, 2013).

### EMOTIONAL DISTRESS

The [National Comprehensive Cancer Network (NCCN), 2003]; http://www.nccn.org established in 1997 a multidisciplinary team, consisting of health care professionals from different fields that worked on the first set of clinical practice standards and guidelines for the assessment and management of the psychosocial consequences of cancer (Holland, 1997). The panel developed a specific instrument, the distress thermometer (DT), as a short screening instrument to routinely and rapidly assess distress in cancer settings. The word “distress” was chosen to define “*a multifactorial unpleasant emotional experience of a psychological (cognitive, behavioral, emotional), social, and/or spiritual nature that may interfere with the ability to cope effectively with cancer, its physical symptoms and its treatment. Distress extends along a continuum, ranging from common normal feelings of vulnerability, sadness and fears to problems that can become disabling, such as depression, anxiety, panic, social isolation and existential and spiritual crisis […].”* The DT is visual analogue tool asking the subject to rate his/her level of distress on a 0–10 scale (from No Distress to Extreme Distress) and to check for possible problems in different areas, including physical, emotional, spiritual, family, and practical problems. National Comprehensive Cancer Network (NCCN), (2003) Distress Management panel published the standards for psychosocial care of cancer patients, establishing a set of quality measures for screening and algorhythms for managing distress and psychiatric disorders (e.g., adjustment disorders, depression, suicide and suicide risk, cognitive disorders) which have been regularly updated on annual basis (Holland et al., 2013). The DT and the related list of possible problems (e.g., physical, practical, interpersonal, spiritual, emotional) increasing the risk for distress have been used worldwide, with data showing that although cut-off scores varied by language, country, and clinical setting and to sample characteristics, in the majority of studies, a score of ≥4 maximize sensitivity and specificity relative to an established criterion (e.g., psychiatric interview, other questionnaires) for psychosocial morbidity (Donovan et al., 2014).

Policies about the routine use of short screening tools, such as the DT or other visual analogue scales (e.g., Edmonton Symptom Assessment Scale, ESAS), have been implemented in several countries, as a way to rapidly identify patients reporting levels of distress indicative of psychosocial morbidity and facilitate their proper referral to psychosocial oncology services (Bultz and Carlson, 2006; Bultz et al., 2011; Holland, 2013). The implications relative to the context and the trajectory of distress, the role of the patients’ perspective (using screening questionnaires, practitioners cannot discover a patient’s thoughts or feelings beyond what the patient already knows and chooses to disclose) and the transfer from a diagnostic to a public health framework for screening are part of a debate still ongoing (Bultz and Johansen, 2011; Salmon et al., 2014).

### ANXIETY AND DEPRESSION

As reported by Mitchell et al. (2011) and Mitchell and Bultz (2014) a number of data have been accumulated regarding the screening for depression and anxiety, with the Depression in Cancer Consensus Group reporting diagnostic validity studies involving at least 19 tools designed to help clinicians identify depression in cancer settings (Mitchell et al., 2012). Two stem questions derived form the Patient Health Questionnaire (PHQ-2), the Beck Depression Inventory (BDI-II) where considered acceptable. Other instruments have been applied in psychosocial oncology and palliative care showing that some tools can be employed as sensitive and specific tools in clinical settings, including the Hospital Anxiety and Depression Scale (HADS; Zigmond and Snaith, 1983). However recent reviews found that the HADS is a suitable tool for initial screening for anxiety and depression (Luckett et al., 2010; Mitchell et al., 2010), but it cannot be proposed as a case-finding (diagnostic) instrument. Screening for anxiety has less been taken into consideration (apart from the data deriving from the HADS), although it is extremely important in cancer settings. The State-Trait Anxiety Inventory (STAI), the Generalized Anxiety Disorder scale (GAD-7), the GAD for DSM, and Fear of Disease Progression Scale (FoP; Herschbach et al., 2005) have been reported as tools to be applied in cancer settings, although their acceptability is also reported to be modest.

### OTHER SYMPTOMS/SYNDROMES AND CLINICAL CONDITIONS

In oncology and palliative care settings, other clinical conditions have been the object of studies aimed at evaluating the feasibility of screening procedures. Cognitive disorders and impairment have been examined in cancer as possible consequences of treatment. Data regarding brief testing (bedside cognitive testing) exist with studies evaluating the sensitivity/specificity of well-known instruments, including the Mini-Mental State Examination (MMSE), which seems to be less accurate than other tools, the Addenbrooke’s Cognitive Assessment – Revised (ACE-R), the abbreviated mental test score (AMTS), and 6-item cognitive impairment test (6CIT; Mitchell and Bultz, 2014).

Data have been also collected regarding the dimensions of QoL and wellbeing. With this respect the literature regarding QoL is extremely vast and besides the scopes of this paper. It suffice to say that several groups, such as the European Organization for Research and Treatment of Cancer (EORTC), have developed instruments to measure a number of areas and items related to QoL, patient’s satisfaction with doctors and health acre profession also in general, physical, and mental symptoms, with modules relative to the single cancer disorders and sites. The EURO-Qol-5, the Health Survey-36 (SF36), the Rotterdam Symptom Check-List (RSCL), the Nottingham Health Profile (NHP), and many others have been however used more for research purposes rather than routine clinical care.

## ASSESSMENT OF BIOPSYCHOSOCIAL DIMENSIONS

Once screened for possible emotional and psychosocial unmet needs or problems, patients showing symptoms or problems should be assessed in a more proper way to examine the structural characteristics of their clinical condition and other psychosocial dimensions.

### COPING AND ATTACHMENT

Assessment of coping, as the pattern of thoughts, beliefs, and behaviors in response to stressful events, is part of a diagnostic approach to cancer patients, since maladaptive styles among cancer patients are intrinsically related to psychopathology (Grassi et al., 1993). According to the transactional theory of stress and coping (indicating that a stressor is initially appraised and, subsequently, the resources available to deal with the stressor are examined by the individual), several measures of coping have been developed (Mitchell and Bultz, 2014). The Mental Adjustment to Cancer (MAC) scale and the shorter Mini-MAC version have been particularly used in cancer and palliative care settings with data showing the good property of the scale (Greer et al., 1989; Watson and Homewood, 2008) in identifying specific styles of coping, including denial/avoidance (i.e., deliberate effort not to think about cancer as a way cope), fighting spirit (i.e., the tendency to see the illness as a challenge), fatalism (i.e., living in the moment and take one day at a time), helplessness/hopelessness (i.e., desperation and hopelessness regarding the illness and the future), and anxious preoccupation (i.e., the tendency to be anxious and extremely preoccupied about the illness).

Attachment style is also a significant area to be explored in the assessment phase both in oncology and palliative care, since the way in which the patient has experienced early relations with caregiving figures in the past relates to her view of herself and to the expectation she has from the health care provider (or health care system; Grassi et al., 2014). In medically ill patients, Maunder and Hunter (2009, 2012) have described four possible patterns of attachment, namely secure, preoccupied, dismissing, and fearful, which are related to illness behavior, health care relationships, and health outcomes (Hunter and Maunder, 2001). In the setting of palliative care, Tan et al. (2005) have described the importance of attachment processes as critically important determinants of therapeutic relationships, particularly when the aims of clinicians are to improve the QoL of patients and to address the suffering that encompasses the physical, psychosocial, and spiritual realms of individuals’ and families’ experiences with terminal illness. In research with patients with end-stage cancer, attachment anxiety, and avoidance have been found to be associated with less social support and more psychological distress (Hunter et al., 2006). A further study of patients with metastatic gastrointestinal and lung cancer has found that both the Experiences in Close Relationships scale (ECR) in its 36-item version and in a shorter form (16 items) are valid tools to identify several dimensions of attachment in oncology (e.g., four first-order factors Worrying about Relationships, Frustration about Unavailability, Discomfort with Closeness, Turning Away from Others and two second-order factors, Attachment Anxiety and Avoidance; Lo et al., 2009).

### DEMORALIZATION, HEALTH ANXIETY, AND OTHER PSYCHOSOCIAL DIMENSIONS

The assessment of other psychosocial dimensions among cancer patients has been raised by research in oncology (Grassi et al., 2007a). The Diagnostic Criteria for Psychosomatic Research (DCPR; Fava et al., 1995) consists of twelve clinical clusters which explore a variety of possible psychological conditions and emotional responses to medical illness. Four clusters are related to patients’ ways of perceiving, experiencing, evaluating, and responding to their health status (abnormal illness behavior: disease phobia, thanatophobia, health anxiety, and illness denial); four clusters are related to the concept of somatization (i.e., functional somatic symptoms secondary to psychiatric disorders, persistent somatization, conversion symptoms, and anniversary reaction); and four to psychological dimensions that have been frequently and consistently found in medical patients (i.e., alexithymia, type A behavior, irritable mood, and demoralization; Sirri et al., 2007). The DCPR constructs of demoralization, alexithymia, irritable mood, and anniversary reaction were found as significant dimensions (not detectable by ICD and DSM nosographic systems) to be taken into account in medically ill patients (Mangelli et al., 2005, 2006; Grassi et al., 2007b; Guidi et al., 2011; De Vries et al., 2012; Fava et al., 2012; Porcelli et al., 2012, 2013). In oncology settings, health anxiety (37.7%), demoralization (28.8%), and alexithymia (26%) were the most frequent DCPR clusters reported by cancer patients in different phases of illness (Grassi et al., 2004, 2005). Patients with DCPR syndromes reported higher levels of sadness, more physical symptoms, poorer well-being, poorer leisure activity, and lower support from interpersonal ties, than women without any DCPR syndrome, with higher scores on the assessment of worries and preoccupation related to cancer (e.g., the illness itself, the effects of treatment, feeling different from others, the impact on sexual life, the future). Other dimensions related to the concept of demoralization, as a clinical syndrome separated from major depression, have been shown to extremely important in oncology. Loss of meaning and hope can determine a sense of worthlessness on one’s own life and in the future which is the hallmark of demoralization, as a syndrome to be measured in oncology settings (Angelino and Treisman, 2001; Kissane et al., 2001). A number of recent studies have shown that demoralization in cancer patients is related to due to confrontation with existential stressors that, throughout the illness across all disease stages, impair the sense of mastery and competence. While global sense of meaning is an important protecting factor regarding the development of demoralization and distress symptoms (Vehling et al., 2011), loss of dignity also has been found to be related to demoralization (Vehling and Mehnert, 2014).

Abnormal illness behavior (i.e., affective inhibition, disease conviction in spite of medical reassurance, frictions in interpersonal relationships) and somatization (i.e., the tendency to evaluate in somatic terms bodily functions) have been also reported by both cross-sectional (Grassi et al., 1989; Chaturvedi et al., 1993) and prospective studies of cancer patients (Grassi and Rosti, 1996). The importance of assessing this dimension has been undelined by some authors (Chaturvedi et al., 2006; Grassi et al., 2013a), who showed that somatic symptoms may magnify disability resulting from cancer, interfere with treatment adherence and decisions, cause delay in recovery, result in poor outcome and recurrence, and reduce overall well-being and QoL, besides complicating the diagnosis of major depression due to the overlap of symptoms occurring as a result of the underlying disease, depression, or somatoform disorder.

## CROSS-CULTURAL CONSIDERATIONS

A specific topic to be discussed when speaking about screening and assessment of psychosocial consequences of cancer regards the importance of taking into account cross-cultural issues. In fact in the last thirty years, attention has focused on the implications of cultural diversity in clinical settings, particularly for racial and ethnic minorities for whom health disparities are related to socioeconomic disadvantage or the difficulty of integrating their cultural model into the dominant model (Kagawa-Singer et al., 2010; Surbone, 2012). Since cancer and palliative care settings are gradually becoming multiethnic and multicultural, the need for clear policies of screening and assessment which take into account the implications determined by cultural diversity, is nowadays mandatory (Grassi and Riba, 2012).

Language, ethnicity, race, and religion, have an important role in affecting the patients and families perception of illness, in influencing communication and doctor–patient relationship (e.g., disclosure of information related to diagnosis and prognosis, role of patient and family in decision-making). Culture may also influence a patient’s coping mechanisms, including psychological response to a cancer diagnosis, the presence, or absence of psychopathological disorders (e.g., phenomenology of anxiety or depression, abnormal illness behavior, somatization), the awareness and knowledge of treatment options, and their acceptance of psychological intervention. All these phenomena should be taken into consideration when training physicians and multidisciplinary oncology and palliative care teams (Grassi et al., 2015).

For these reasons, instruments and tools should be translated and adapted according to the different languages and cultures, and accuracy and analysis of validity and reliability of these tools should be also considered in order to be clinically useful to the clinician.

As an example, the US-based NCCN practical guidelines for routinely screening, assessing, and managing distress are today one of the most significant points of reference in psychosocial oncology (Holland et al., 2013), but the authors may not have anticipated the potential utility of these guidelines in other cultures. The translation, adaptation, and application of the NCCN guidelines in a number of different countries and cultures indicated the need for these guidelines to take into account the specific needs of different health care systems, and relative cultures, in the different countries (Donovan et al., 2014). This is extremely important since the attitudes toward the concepts of illness and suffering, decisions about treatment, and the whole of oncology care are framed by cultural factors that also influence the social structure of doctor–patient relationship within which both screening and assessment of psychosocial needs are part of. Although it is said that research on the impact of cultural issues in oncology is not well-developed, data have accumulated regarding the importance of cultural variables in cancer care and the specific role of cultural competence in providing care (Seeleman et al., 2009). Cultural (and linguistic) competence as a set of congruent behaviors, attitudes, and policies enabling effective work in cross-cultural situations, is thus a specific role in oncology, where competence implies having the capacity to function effectively as an individual and an organization within the context of the cultural beliefs, behaviors, and needs presented by patients and their support system (Surbone, 2004).

As another example, a study of breast cancer survivors of different backgrounds (i.e., African American, Asian American, Latina, and Caucasian) it was shown that psychosocial concerns related to worry about children and burdening the family, body image, and sexual health concerns, beliefs about illness, gender role, family obligations (e.g., self-sacrifice), as well as language barriers were significantly different among the different cultural groups (Ashing-Giwa et al., 2004). In another study, researchers demonstrated that immigrant Chinese breast cancer survivors may express symptoms in culturally unique ways (e.g., hot-cold imbalances) and may be at higher risk for distress compared with US-born Chinese and non-Hispanic breast cancer survivors, because of cultural norms that influence the tendency to express one’s own needs to physicians or to challenge physicians when one’s own needs are not met (Wang et al., 2012).

These data confirm the need for cultural sensitivity and competence of cancer care providers. It is clear that the creation and the dissemination of true patient/family-centered care favor cultural competence. In culturally sensitive patient/family-centered care, the clinical encounter is grounded in communication whereby cultural cues (i.e., values and beliefs) of the patient and the clinician are incorporated within the therapeutic relationship and mutually shared (Kumagai and Lypson, 2009; Teal and Street, 2009; Surbone, 2010).

Thus, screening and assessment should be part of a specific encounter with a cancer patient and her family, and thus culturally competent communication should be part of a more effective patient-centered communication framework, with cultural competence as a necessary component in establishing a relationship, gathering information, assessing, and managing patients’ problems, including psychosocial disorders.

## CONCLUSION

Given the importance of psychological disorders secondary to cancer diagnosis and treatment, careful examination of symptoms and psychosocial needs is mandatory in oncology and palliative care settings. Since proper psychiatric evaluation of all patients is impossible, guide-liens have been implemented with the aim of facilitating procedures of screening for distress and psychosocial symptoms/needs as a routine good clinical practice. Assessment is considered as a second step approach for those presenting detectable symptoms/needs. Several dimensions, besides those emerging form a standard psychiatric interview, should be specifically considered. In fact, while the main aims of a standard psychiatric approach are to establish whether a psychiatric disorder or other condition requiring clinical psychiatric/psychosocial attention is present and to collect data to support the differential diagnosis and a comprehensive clinical formulation [American Psychiatric Association (APA), 2006], there are also different levels of diagnosis that should be considered as not mutually exclusive but integrated in consultation psychiatry/psychosomatic medicine and, by extension psychosocial oncology: the clinical diagnosis, which it is nosologically-oriented, allowing clinicians to communicate with one another about the signs and symptoms the patient is presenting (e.g., DSM, ICD); the dynamic-interpersonal diagnosis, which is interpersonally-oriented and includes the psychological and social variables (or forces) involved in the presentation of the patient’s symptoms, vulnerabilities and strengths; and the genetic diagnosis which is historically-oriented, including early experiences and life-events (e.g., attachment early experiences), coping and social support (Wise, 1986). These approaches, in the specific setting of cancer and palliative care, as well as of consultation-liaison psychiatry and psychosomatic medicine (Smith et al., 2011), are part of the process of assessment and should consider multiple dimensions. It is in fact necessary to understand the many different variables, such as the factors affecting individual vulnerability (e.g., life events, chronic stress and allostatic load, well-being, and health attitudes) and the psychosocial correlates of medical disease (e.g., psychiatric disturbances, psychological symptoms, illness behavior, and QoL) which are possibly implicated not only in “classical” psychiatric disorders but more broadly in psychosocial suffering (Fava, 1996; Fava and Sonino, 2005). Thus, the role of screening for distress (e.g., DT, ESAS, and other psychometric questionnaires), associated with more specific assessment of other psychosocial dimensions related to cancer diagnosis and treatment (e.g., attachment, coping, DCPR) need to be considered as part of routine care in oncology and palliative care settings (Grassi et al., 2013b).

## Conflict of Interest Statement

The authors declare that the research was conducted in the absence of any commercial or financial relationships that could be construed as a potential conflict of interest.
